# Lymphedema of the Breast Following Partial Mastectomy and Oncoplastic Reduction

**DOI:** 10.3390/lymphatics4020022

**Published:** 2026-04-22

**Authors:** Shahnur Ahmed, Dylan Roth, Luci A. Hulsman, Rachel M. Danforth, Ravinder Bamba, Kandice K. Ludwig, Mary E. Lester, Karl Y. Bilimoria, Carla S. Fisher, Aladdin H. Hassanein

**Affiliations:** 1 Division of Plastic Surgery, Department of Surgery, Indiana University School of Medicine, Indianapolis, IN 46202, USA; 2 Division of Breast Surgery, Department of Surgery, Indiana University School of Medicine, Indianapolis, IN 46202, USA; 3 Division of Surgical Oncology, Department of Surgery, Indiana University School of Medicine, Indianapolis, IN 46202, USA

**Keywords:** breast lymphedema, oncoplastic reduction, partial mastectomy, closure technique, wise pattern, vertical, no-vertical

## Abstract

Breast lymphedema is characterized by skin thickening/swelling of the breast and is common following partial mastectomy and radiation. Oncoplastic reduction performed during partial mastectomy removes additional breast tissue compared to partial mastectomy alone to optimize breast contour. Recent literature has suggested oncoplastic reduction in patients with macromastia undergoing breast-conservation surgery is protective of breast lymphedema, decreasing rates from 11% to 3%. The purpose of this study is to assess the rates of breast lymphedema after partial mastectomy and oncoplastic reduction and identify risk factors. A single-center retrospective study was performed of breast cancer patients following partial mastectomy and oncoplastic reduction (2018–2023). Patients underwent contralateral breast reduction for symmetry. Breast lymphedema was assessed. Demographics data and risk factors were evaluated. This study included 158 patients who underwent partial mastectomy and oncoplastic reduction. Breast lymphedema incidence was 3.2% (5/158). Including contralateral non-cancerous breast symmetry reduction, lymphedema occurred in 3.6% (5/140) of irradiated breasts and 0% (0/176) of non-irradiated breasts (*p* = 0.0164). Among irradiated breasts, skin necrosis occurred in 11.4% (16/140) compared to 4.5% (8/176) of non-irradiated breasts (*p* = 0.031). Breast lymphedema developed 207.4 ± 37.6 days postoperatively and 101.6 ± 15.9 days following adjuvant radiation. Mean follow-up was 639 days. Breast lymphedema incidence following partial mastectomy and oncoplastic reduction was 3.6% in this series and occurs 3–4 months after radiation. Radiation was the only significant risk factor for developing breast lymphedema. This largest series on breast lymphedema after oncoplastic reduction corroborates that oncoplastic reduction may be protective.

## 1. Introduction

Lymphedema of the breast following breast-conserving therapy is clinically characterized by thickening and swelling of the breast skin [1,2]. Breast lymphedema may occur from lymphatic disruption, leading to breast pain, recurrent cellulitis, and overall poorer quality of life [2–4]. Transient breast edema has not been well-distinguished from breast lymphedema, which further contributes to a limited understanding of this disease process [2,5]. Adjuvant radiation to the breast has been identified as an independent risk factor of developing breast lymphedema [6–8]. Unlike breast cancer-related upper extremity lymphedema, breast lymphedema is poorly characterized, with reported rates between 5 and 94%, and has even more limited treatment options [2,9,10]. Non-operative management of breast lymphedema is adapted from breast cancer-related lymphedema (BCRL) of the arm, which includes chest compression wrapping and elastic therapeutic tape application [6–8,11–16]. There is no cure for breast lymphedema, and unlike BCRL, there are no well-characterized operations to improve or prevent the condition [2,7,17–25].

Oncoplastic breast surgery may be offered to early-stage breast cancer patients who undergo breast-conserving therapy to remove cancerous breast tissue and optimize breast cosmesis [26]. Oncoplastic reduction removes additional breast tissue compared to partial mastectomy (PM) alone to maintain breast form and symmetry [26–35]. The approaches involve additional scars and dissection, which can possibly disrupt dermal lymphatics [2,36]. The incidence of breast lymphedema after PM with oncoplastic reduction is not well reported [1,2]. Recent literature has suggested oncoplastic reduction in patients with macromastia undergoing breast-conservation surgery is protective of lymphedema, with a rate of 3% for patients undergoing partial mastectomy with oncoplastic reduction compared to 11% for patients that had partial mastectomy alone [6]. The purpose of this study is to assess the rates of breast lymphedema after partial mastectomy and oncoplastic reduction and identify risk factors for breast lymphedema.

## 2. Results

In our study, there were 158 patients (316 total breasts) who underwent partial mastectomy with oncoplastic breast reduction (Table 1). Five patients (3.2%, 5/158) developed breast lymphedema, and there were 153 patients (96.8%, 153/158) who did not develop breast lymphedema. There was one patient who underwent a breast ultrasound that demonstrated transient breast edema. The mean age of patients who developed breast lymphedema was 48.8 ± 6.6 years compared to 55.4 ± 9.2 years in those who did not develop breast lymphedema (*p* = 0.117). The average BMI of those who developed breast lymphedema was 36.5 ± 5.8 kg/m^2^ compared to 32.9 ± 7.5 kg/m^2^ in those who did not develop breast lymphedema after partial mastectomy and oncoplastic reduction (*p* = 0.294). Adjuvant whole breast radiation was performed in 100% (5/5) of patients who developed breast lymphedema compared to 88.2% (135/153) in those who did not develop breast lymphedema (*p* = 1).

Breast lymphedema did not occur in the contralateral, non-cancerous breasts that were reduced for symmetry (Table 2). Breast lymphedema occurred in 3.6% (5/140) of breasts that underwent radiation and did not occur if the breast was not irradiated (0/176) (*p* = 0.0164) when the contralateral breast reduction was included. Oncoplastic reduction using Wise pattern incisions was performed in 60% (3/5) of patents who developed breast lymphedema compared to 52.9% (81/153) in those who did not develop breast lymphedema (*p* = 1). Vertical technique incisions for oncoplastic reduction were used in 40% (2/5) of breast lymphedema patients compared to 35.9% (55/153) in patients who did not develop breast lymphedema following partial mastectomy and oncoplastic reduction (*p* = 1). The remaining 11.2% (17/153) of non-breast lymphedema patients underwent no-vertical modified-Robertson incisions for oncoplastic reduction, and none developed breast lymphedema. Sentinel lymph node (SLNB) biopsy was performed in 80% (4/5) of breast lymphedema patients compared to 83.7% (128/153) in those who did not develop breast lymphedema (*p* = 1). Axillary lymph node dissection (ALND) was not performed in patients who developed breast lymphedema compared to 5.2% (8/153) in those (*p* = 1) who did not develop lymphedema of the arm or breast.

Skin necrosis occurred in 0% (0/5) of breast lymphedema patients compared to 15.6% (24/153) of those who did not develop breast lymphedema (*p* = 1). Surgical-site infection occurred in 2.9% (4/140) of irradiated breasts compared to 0.6% (1/176) of non-irradiated breasts. No patients who had breast lymphedema developed skin necrosis or surgical-site infection. Breast lymphedema was clinically diagnosed 207.4 ± 37.6 days postoperatively and 101.6 ± 15.9 days after completion of adjuvant radiation (Table 3). Management of all patients who developed breast lymphedema included outpatient physical therapy, daily breast massage, and chest wall elastic therapeutic tape. The mean follow-up was 639.0 ± 282.9 days (range 378–1727 days).

## 3. Discussion

Breast lymphedema is distinct from breast cancer-related lymphedema of the arm and characterized by persistent breast swelling that may lead to breast pain and recurrent cellulitis [1,2]. Radiation has been identified as an independent risk factor for the development of breast lymphedema [2,12,15,16]. A study of 64 patients with macromastia who underwent partial mastectomy and oncoplastic reduction found that oncoplastic reduction is protective from breast lymphedema by 3.7-fold (3% vs. 11%) when compared to patients who had partial mastectomy alone [6]. This largest series on breast lymphedema after oncoplastic reduction corroborates that oncoplastic reduction may be protective. While studies have been performed to describe breast lymphedema after partial mastectomy, there is limited information assessing those who have breast lymphedema following PM and oncoplastic reduction.

In this study, the incidence of breast lymphedema was 3.2% overall and 3.6% among total breasts irradiated following PM and oncoplastic reduction with at least 1-year follow-up. Among all patients who received breast-conserving therapy, previous literature has attempted to describe breast lymphedema incidence, with reported rates from 5 to over 90% [2,5,9]. The reasons for a large range of incidence include the lack of distinguishing breast skin radiation changes from breast lymphedema, limitations in utilizing imaging modalities (ultrasound, magnetic resonance imaging, indocyanine green lymphangiography) to diagnose breast lymphedema, and patient reporting of persistent breast swelling leading to breast lymphedema diagnosis, which may be subjective [2]. The diagnosis of breast lymphedema is not standardized [2–5,9]. The use of imaging to diagnose breast lymphedema varies across institutions [2–5,9]. Variation in the use of imaging to diagnose breast lymphedema is a limitation of our study and may underestimate the incidence of breast lymphedema when not used. A prospective study of 836 patients with 2.3-year follow-up identified oncoplastic surgery as a risk factor for breast edema in those who underwent breast-conserving surgery [4]. The rate of breast lymphedema was 5.5% at 18-months postoperatively [4]. In 58% of patients who received oncoplastic surgery, the actual oncoplastic technique used was not characterized [4]. Our study may contribute to a breast lymphedema incidence of 3.6% in the largest series that is more representative of breast cancer patients who undergo a formal oncoplastic breast-reduction technique following partial mastectomy and radiation.

A prospective study of 144 patients identified that incision location was a risk factor using odds ratios (OR) for breast lymphedema [36]. Patients with incisions located in the lower inner quadrant (OR 11.49), upper outer quadrant (OR 4.67), or centrally (OR 9.40) had higher odds of developing breast lymphedema compared to those who had incisions in the lower outer quadrant [36]. However, the study did not assess patients who received oncoplastic breast reduction [36]. Wise pattern incisions result in the most scars with periareolar, vertical and IMF [37,38]. The vertical technique utilizes incisions located centrally (periaerolar and vertical incision) and is based on a superomedial or medial pedicle [30,32,39]. While not statistically significant in this study, patients who received oncoplastic surgery with closure using a no-vertical scar technique did not develop breast lymphedema. A previous study of 144 patients who underwent breast reduction theorized that centrally located incisions may potentially disrupt breast lymphatics, theoretically increasing breast lymphedema risk [36]. A no-vertical scar method minimizes scars, which includes a periareolar incision and IMF [40]. Further evaluation of oncoplastic closure technique and incision location may be warranted to highlight breast lymphedema occurrence in these patients.

Adjuvant radiation to the breast was assessed in our study. The incidence of breast lymphedema among irradiated breasts was 3.6% at 1-year follow-up and was more common compared to non-irradiated contralateral breasts in this study. Breast lymphedema was clinically diagnosed at 101 days following adjuvant whole breast radiation in our study. Previous reports have demonstrated that radiation is a significant risk factor of developing breast lymphedema and may occur as early as 3 months after completing radiation [2,4,12,41]. The findings from our study are consistent with reported literature on the timing of breast lymphedema following breast-conserving therapy and may be used for patient counseling in those who receive oncoplastic breast reduction. Our study has limited generalizability given its retrospective, single-center design and small sample size. On post hoc power analysis, our study had over 90% power to detect a 5% difference based on our effect size. A surgical approach with partial mastectomy alone was not assessed in our study and is a study limitation.

## 4. Methods

A single-center retrospective review was performed of breast cancer patients who underwent partial mastectomy and oncoplastic breast reduction between 2018 and 2023. Patients underwent contralateral breast reduction for symmetry. Breast lymphedema was assessed and diagnosed clinically based on physical examination findings of edema focally affecting the breast skin (peau d’orange) and symptoms of breast heaviness or discomfort that was persistent for greater than 3 months.

Variables recorded included patient demographic information, body mass index (BMI), diabetes, smoking status, breast cancer stage, type of axillary node surgery (sentinel node, axillary dissection), chemoradiation therapy, and technique for oncoplastic surgery. Breast lymphedema, timing onset of breast lymphedema following adjuvant radiation, postoperative complications (e.g., skin necrosis, surgical-site infection), and mean follow-up time were variables of interest.

**Figure 1.**
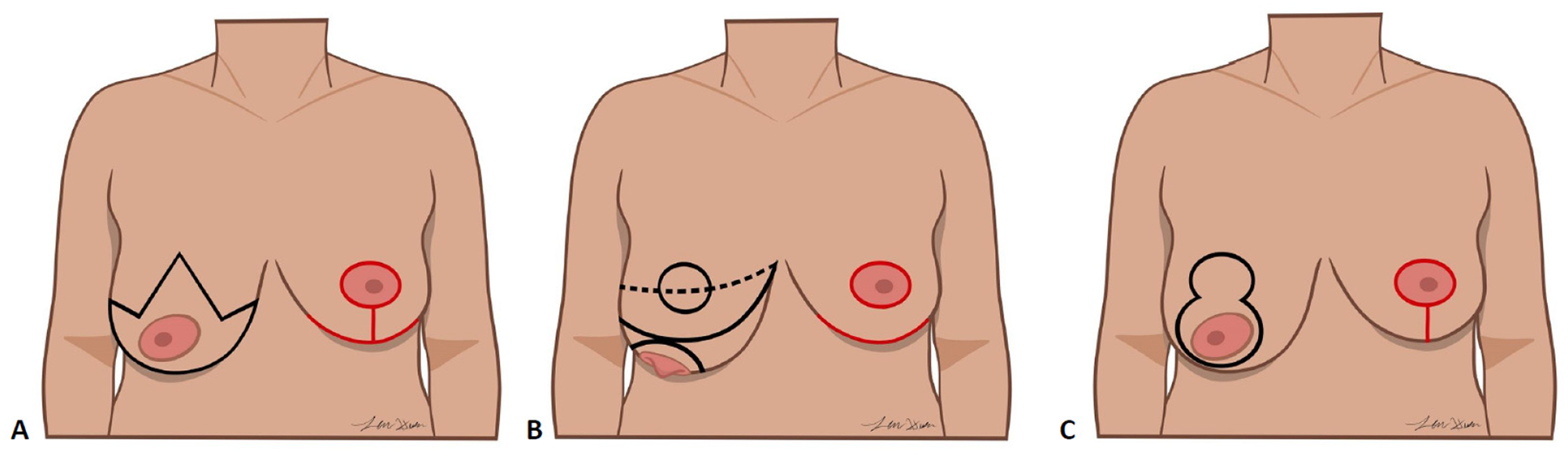
Oncoplastic breast-reduction techniques. The oncoplastic method was chosen based on tumor location and patient anatomy. (**A**) The Wise pattern typically uses an inferiorly based pedicle to perfuse the nipple and results in periareolar, vertical and inframammary fold scars. (**B**) A no-vertical scar technique may be performed using an inferior pedicle to maintain prefusion to the nipple–areolar complex and avoids introduction of a vertical scar. (**C**) A vertical pattern commonly utilizes the medial or superomedial breast pedicle and results in a periareolar and vertical scar.

Patients were candidates for oncoplastic reduction if PM would likely result in significant asymmetry or contour abnormality or those with symptoms of macromastia. The techniques used to perform oncoplastic breast reduction were the Wise pattern, vertical incision, and no-vertical scar technique (Figure 1) [40]. The oncoplastic method was chosen based on tumor location and patient anatomy. The Wise pattern typically uses an inferiorly based pedicle to perfuse the nipple and results in periareolar, vertical and inframammary fold (IMF) scars [37,38]. A vertical pattern commonly utilizes the medial or superomedial breast pedicle and results in a periareolar and vertical scar [30,32,39,42]. A no-vertical scar technique, also referred to as the modified Robertson, may be performed using an inferior pedicle to maintain prefusion to the nipple–areolar complex and avoids introduction of a vertical scar [21,40].

Statistical analyses were performed using IBM SPSS Statistics Version 29 (IBM Corporation; Armonk, NY, USA). Categorical variables were analyzed using the Fisher exact test. Continuous variables were compared using independent-samples *t* tests. Two-tailed values of *p* < 0.05 were considered statistically significant.

## 5. Conclusions

The incidence of breast lymphedema following partial mastectomy with oncoplastic reduction was 3.2% in the largest series and occurred, on average, 3–4 months following radiation in this study. The findings on breast lymphedema incidence and timing from this study after PM and oncoplastic reduction may be used for patient counseling. Radiation was the only significant risk factor for developing breast lymphedema. No breast lymphedema occurred in patients that had a no-vertical scar modified-Robertson approach. This largest series on breast lymphedema after oncoplastic reduction corroborates that oncoplastic reduction may be protective.

## Figures and Tables

**Table 1.** Baseline characteristics of breast cancer patients following partial mastectomy and oncoplastic reduction. ALND: axillary lymph node dissection, DCIS: ductal carcinoma in situ, IDC: invasive ductal carcinoma, ILC: invasive lobular carcinoma, SLNB: sentinel lymph node biopsy. *p*-values < 0.05 were considered statistically significant).

Variables	Group 1 (Breast Lymphedema) (n = 5 Patients)	Group 2 (No Breast Lymphedema) (n = 153 Patients)	*p*-Value
Average age (years)	48.8 ± 6.6	55.4 ± 9.2	0.117
Body mass index (kg/m^2^)	36.5 ± 5.8	32.9 ± 7.5	0.294
Diabetes mellitus	0 (0%)	20 (13.1%)	1
Smoker	1 (20%)	11 (7.1%)	0.3298
Neoadjuvant chemotherapy	0 (0%)	26 (16.9%)	0.5919
Adjuvant chemotherapy	0 (0%)	43 (28.1%)	0.3242
Radiation			
Number of Irradiated breasts	5	135	-
Number of Non-irradiated breasts	5	171	-
Adjuvant radiation	5 (100%)	135 (88.2%)	1
Prior radiation	0 (0%)	9 (5.9%)	1
Breast Cancer Type			
DCIS	3 (60%)	36 (23.5%)	0.0968
IDC	2 (40%)	97 (63.4%)	0.3628
ILC	0 (0%)	11 (7.2%)	1
Mixed	0 (0%)	7 (4.6%)	1
Other	0 (0%)	2 (1.3%)	1
Clinical Breast Cancer Stage			
0	3 (60%)	38 (24.8%)	0.1103
1	2 (40%)	64 (41.8%)	1
2	0 (0%)	45 (29.4%)	0.3225
3	0 (0%)	6 (3.9%)	1
Type of Oncoplastic Reconstruction			
Wise pattern	3 (60%)	81 (52.9%)	0.3751
Vertical technique	2 (40%)	55 (35.9%)	1
Modified Robertson	0 (0%)	16 (10.4%)	1
Type of Axillary Surgery			
None	1 (20%)	13 (8.5%)	1
SLNB only	4 (80%)	128 (83.7%)	1
SLNB with immediate ALND	0 (0%)	1 (0.6%)	1
SLNB with delayed ALND	0 (0%)	3 (2.0%)	1
ALND only	0 (0%)	4 (2.6%)	1
Skin necrosis	5 (0%)	24 (15.7%)	1

**Table 2.** Outcomes of breast cancer patients following partial mastectomy and oncoplastic breast reduction based on radiation exposure.

Variables	Irradiated Breasts (n = 140)	Non-Irradiated Breasts (n = 176)	*p*-Value
Breast lymphedema	5 (3.6%)	0 (0%)	0.0164 *
Surgical-site infection	4 (2.9%)	1 (0.6%)	0.1747
Tumor resection weight (g)	561.8 ± 663.7	904.9 ± 610.1	0.2338

*p*-values <0.05 were considered statistically significant (*).

**Table 3.** Timing to diagnosis of breast lymphedema patients.

Variables	Breast Lymphedema (n = 5 Patients)
Incidence	3.2% (5/158)
Time to diagnosis postoperatively	207.4 ± 37.6 days
Time to diagnosis after radiation	101.6 ± 15.9 days

## Data Availability

The data supporting the findings are available from the corresponding author upon reasonable request.
